# Uncovering the genetic basis of competitiveness and the potential for cooperation in plant groups

**DOI:** 10.1098/rspb.2024.1984

**Published:** 2025-03-12

**Authors:** Jay M. Biernaskie, Gina A. Garzón-Martínez, Fiona M. K. Corke, John H. Doonan

**Affiliations:** ^1^ Department of Crop Genetics, John Innes Centre, Norwich, UK; ^2^ Institute of Biological, Environmental, and Rural Sciences, Aberystwyth University, Aberystwyth, Ceredigion, UK; ^3^ AGROSAVIA, Mosquera, Colombia

**Keywords:** *Arabidopsis thaliana*, competition, Hamiltonian agriculture, MAGIC lines, multi-parent populations, quantitative trait loci

## Abstract

Crop productivity was transformed by incorporating dwarfing genes that made plants smaller and less competitive (more cooperative). Beyond such major shifts in plant size, however, it is not clear how much variation in competitiveness remains and how to find its genetic basis. We performed plant density experiments, using 484 lines of the *Arabidopsis thaliana* multi-parent advanced generation inter-cross population, to compare methods for mapping the genetic basis of plant competitiveness. We first found that a major dwarfing gene, the *erecta* allele, caused reduced competitiveness and higher group productivity. Then, measuring competitiveness more generally, we found: (i) extensive variation in generic measures of competitiveness that extended beyond the effects of the *erecta* allele; (ii) a novel genomic region underlying variation in competitiveness; and (iii) that some measures of competitiveness were more useful than others. Our results show how modern genomic resources, including multi-parent populations, could uncover hidden genes for more cooperative crop plants.

## 1. Introduction

The traits of a plant can help or harm its neighbours, and these ‘social’ traits should have a major influence on group productivity, including crop yield. A competitive (or selfish) trait is good for the individual expressing the trait but harmful to its neighbours [1,2]. More competitiveness in the group therefore leads to more harm and lower total productivity—a ‘tragedy of the commons’ [3–5]. To counter this, plant breeders could favour reduced individual competitiveness as a form of cooperation among plants [6–9].

Some obvious social traits have been adopted with great success in plant breeding and agriculture. In dense crops, group productivity may be promoted by using smaller plants that take fewer resources away from their neighbours (e.g. plants with shorter stature or smaller leaves). The key example comes from historic yield increases during the Green Revolution, when shorter (dwarfed) rice and wheat types were favoured, at least in part, because they are less competitive than taller types [10–12]. Later, genetic studies revealed that dwarfism was caused by major genes with large effects [13,14].

However, beyond obvious traits like dwarfism, it is not clear how much variation in competitiveness remains and how to find its genetic basis. Rather than looking for specific plant traits, a promising approach is to measure competitiveness itself in a wide diversity of genotypes and then map this generic trait to the genome [15–17]. The challenge is to find variation in competitiveness that is separate from variation among genotypes in their overall productivity in an experimental environment. Early studies used simple competition indices, based on individual plant performance in low- versus high-density monoculture groups [15,16]. More recently, Wuest *et al*. [17] introduced a method for quantifying an individual-versus-group tradeoff, measuring a genotype’s group performance in monocultures and its individual performance in mixtures of several ‘tester’ genotypes. This tradeoff approach is an appealing way of finding variation in competitiveness, but replicating a great number of mixed-genotype groups may be impractical for plant breeders.

Here, we use a simple plant density experiment to compare several methods for uncovering the genetic basis of plant competitiveness. We examined within-genotype competition using the *Arabidopsis thaliana* multi-parent advanced generation inter-cross (MAGIC) population, a genomic resource that captures a huge diversity of genetic variation and enables precise mapping of quantitative trait loci (QTLs) [18]. We first mapped the genetic basis of a known dwarfing mutation in the MAGIC population and examined its effect on individual and group productivity. We then measured competitiveness more generally, using competition indices and a simplified individual-group tradeoff analysis. Our aim was to examine (i) the extent of variation in competitiveness beyond the effects of dwarfing; (ii) the genetic basis of competitiveness in general; and (iii) the usefulness of different measures of competitiveness.

## 2. Methods

### (a) Overview

We performed all experiments in the greenhouses of the National Plant Phenomics Centre in Aberystwyth, Wales. In our main experiment (MAGIC experiment), we used 484 recombinant inbred lines from the *Arabidopsis* MAGIC population. Each line was replicated three times in two levels of within-genotype competition: a single plant per pot (‘isolation’ treatment) and four plants of the same genotype per pot (‘group’ treatment). We measured plant height and counted the number of fertile fruits (siliques) as a measure of individual and group performance. To measure the individual competitiveness of each MAGIC line, we used the average silique production in isolation and in groups to calculate competition indices and to quantify an individual-group tradeoff. We then used QTL mapping to examine the genetic basis of plant height and generic measures of competitiveness. In a separate experiment (ERECTA experiment), we used isogenic tall and dwarfed lines to examine the effect of the *erecta* dwarfing mutation on individual and group productivity. We used JMP Version 17 (SAS Institute, Cary, NC) for most statistical analyses, unless otherwise noted.

### (b) Plant material and growing conditions

*Arabidopsis thaliana* (thale cress) is a small annual plant in the mustard family (Brassicaceae). Individuals first grow as a leafy rosette near to the ground and then produce a primary stem and (often) several lateral branches with self-fertilizing flowers and small leaves. *Arabidopsis* is native to Eurasia and Africa, and the seeds of many local populations (accessions) from across this range have been collected and curated.

We obtained all seeds for our experiments from the European Arabidopsis Stock Centre (NASC). For the MAGIC experiment, we used 484 recombinant inbred lines from a core collection of the *A. thaliana* MAGIC population (NASC: ID N782242). For the ERECTA experiment, we used the dwarfed Landsberg *erecta* accession, Ler-0 (NASC ID: NW20), and its tall isogenic line, LER (NASC ID: N163), which is derived from Ler-0 but made homozygous for the wild-type *ERECTA* allele.

The *Arabidopsis* MAGIC population is derived from 19 diverse parental accessions of *A. thaliana*, as described by Kover *et al*. [18]. Genomes of the 19 parents were mixed over four generations of intermating, and near-homozygous inbred lines were then created over several generations of inbreeding. These recombinant inbred lines were genotyped with 1260 single nucleotide polymorphisms; consequently, the genome of each line can be reconstructed as a mosaic of the parental accessions, allowing for precise QTL mapping. For our purposes, it is useful that one of the parental accessions is Ler-0, which carries the *erecta* dwarfing mutation. We identified MAGIC lines carrying this mutation by scoring for the *erecta* phenotype (compact inflorescence and sword-shaped siliques).

We used the same basic growing conditions for all experiments. Seeds were initially sown in individual pots and stratified for 28 days at 5°C with a 16 h day/8 h night photoperiod. Seedlings were then transplanted into 6 cm diameter pots filled with vermiculite on the bottom half and 30% grit sand and 70% Levington F1 peat-based compost on the top half. In pots assigned to a group treatment, we arranged seedlings 1 cm apart. Pots were transferred onto the PlantScreen Phenotyping System (Photon Systems Instruments, Drásov, Czechia) and grown with a 14 h day/10 h night photoperiod, temperatures of 18°C day/15°C night and daily computer-controlled weighing and watering to a constant weight. When plants were done flowering, we transferred them to a neighbouring glasshouse compartment to complete their maturation (all conditions stayed the same, except that we watered pots from below).

### (c) MAGIC experiment

#### (i) Experimental design

We divided the MAGIC experiment into three independent batches that were run consecutively. Each batch contained 160–163 MAGIC lines replicated three times in the isolation treatment and three times in the group treatment. Batches were arranged into three blocks representing different sections of the greenhouse. Each block contained one isolation pot and one group pot for every MAGIC line in the batch, and pots were randomly arranged within the block.

#### (ii) Generic measures of individual competitiveness

In the main text, we consider a reduced dataset for calculating generic measures of individual competitiveness (analyses using the full dataset are given in the electronic supplementary material). This is because, at the end of the MAGIC experiment, some pots in isolation had a high proportion of aborted (undeveloped) siliques. Specifically, plants with many branches had a higher probability of having aborted siliques than plants with fewer branches (electronic supplementary material, figure S1). This suggested that the largest plants in our experiment may have been limited by resources at the fruit-filling stage; consequently, their relatively low silique number may have been an artifact of our experimental conditions. To avoid this complication, we excluded (i) isolation pots that had two or more branches of aborted siliques and (ii) their paired pots in the group treatment from the same line and same block. This left 361 MAGIC lines with paired isolation and group pots that we used to calculate measures of competitiveness.

As a first step in the analyses that follow, we calculated mean trait values for each MAGIC line in the isolation and group treatments. For each treatment level separately, we fit a linear model predicting the trait value (*Y*) and including the model effects: *Batch*, *Block* and *Line (R*) (where *Line* identifies the MAGIC line and *R* denotes a random effect). We then saved the least-squares (LS) mean of the response variable (*Y*) for each MAGIC line. This yielded mean values for each line that were adjusted for any overall differences between batches and blocks. We used this approach to calculate LS means for silique number and for plant height.

#### (iii) Competition indices

We calculated competition indices using the LS mean per-plant silique production of MAGIC lines in isolation and in groups. As a focal competition index, we used the relative interaction intensity (RII) from Armas *et al*. [19]. Using our data, this index is


$$\begin{array}{l} RII_{silique\;no.}=\left(S_{G}-S_{I}\right)/\left(S_{G}+S_{I}\right) \end{array},$$

where *S*_G_ and *S*_I_ are the LS mean siliques per plant for a line in the group treatment and the isolation treatment, respectively. The RII has a continuous range with defined limits (−1 to +1) and a symmetrical distribution, with negative values indicating competition and positive values indicating facilitation. Increasingly negative values indicate more competitive genotypes that were highly productive in isolation relative to their productivity in groups.

We also examined two related indices from the literature. The first index is a ratio of plant performance in low-density to high-density, often called the ‘response to competition’ (RC) [16,20]. Using our data, this is RC_silique no._ = *S*_I_/*S*_G_. The second index is a difference in plant performance between low-density and high-density, which has been called the ‘adaptation to density’ index (ADI) [15]. Using our data, this is ADI_silique no._ = *S*_I_ – *S*_G_. To be consistent with our other measures of competitiveness, we present the negative values of these indices (–RC and –ADI) so that more-negative values indicate greater competitiveness.

#### (iv) Individual-group tradeoff

We quantified an individual-versus-group productivity tradeoff by adapting the approach of Wuest *et al*. [17]. We first fitted an orthogonal linear regression line with LS mean total silique production in groups as the response variable and LS mean silique production in isolation as the predictor variable (assuming equal variances in the response and predictor). This relationship is expected to be positive because some genotypes will tend to grow well in the experimental environment and others will not. The positive slope of this relationship can therefore be considered an axis of ‘vigour’ in our experimental environment. To calculate an individual-group tradeoff score that is independent of vigour, we measured the orthogonal distance of each point from the regression line. Points below the regression line were given negative orthogonal distances (negative tradeoff scores), indicating highly competitive genotypes that were relatively productive in isolation but unproductive in groups. Points above the line were given positive orthogonal distances (positive tradeoff scores), indicating weakly competitive genotypes that were relatively unproductive in isolation but productive in groups.

#### (v) QTL mapping

We used established techniques for QTL mapping in the *A. thaliana* MAGIC population, based on the analytical methods developed by Kover *et al*. [18]. As inputs to the QTL analysis, we used LS mean plant height; all competition indices described above; and the orthogonal distances from the individual-group tradeoff analysis. We performed the QTL analyses with R Statistical Software (v. 4.02.3) using the package *R/qtl2* extended for the analysis of multi-parental populations [21,22]. To establish the statistical significance for a QTL, we used 1000 permutation tests to find an LOD threshold for an experiment-wide Type 1 error rate of 0.05 (QTLs surpassing this threshold were therefore considered statistically significant with *p <* 0.05).

### (d) ERECTA experiment

In the ERECTA experiment, we directly tested the effect of the mutant *erecta* allele on height and silique production, using the same density treatments as the MAGIC experiment. We organized this experiment in seven trays of 15 pots. In each tray, we randomly assigned five pots to the isolation treatment (half of the pots being tall genotypes and half being dwarfed genotypes, on average) and 10 pots to the group treatment (half of the pots tall and half dwarfed). Due to some loss of seedlings, one tray had an incomplete number of pots that we retained in the analysis. Response variables in this experiment were plant height (*Y*_1_) and the total silique production per pot (*Y*_2_). We estimated LS means from linear models including the model effects: *Tray*, *Treatment*, *Genotype* and *Treatment* × *Genotype*. Finally, to check whether dwarfed plants produced fewer seeds/silique, we randomly sampled two siliques from each of 33 Ler-0 plants and 33 LER plants in the group treatment only. Using plant means, we estimated the overall mean seed number per silique for Ler-0 and LER genotypes.

## 3. Results

### (a) Plant height as a competitive trait

#### (i) Genetic basis of plant height

**Figure 1.**
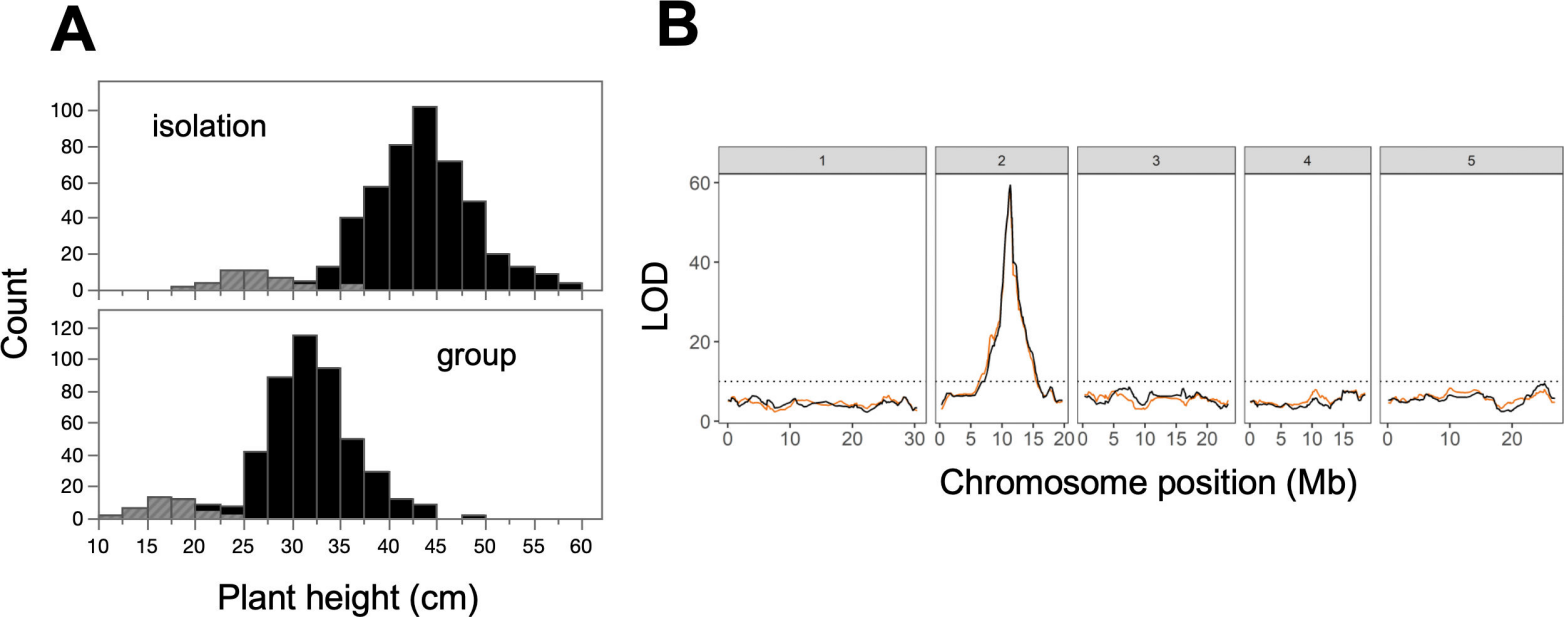
Genetic basis of plant height in the MAGIC population. (A) Variation in plant height, where grey bars indicate MAGIC lines carrying the *erecta* dwarfing allele (*n* = 33 out of 484 lines). (B) QTL mapping for plant height, showing a significant peak near the ERECTA gene on Chromosome 2 (orange line: isolation treatment, black line: group treatment). The LOD score measures the strength of evidence for a QTL at a particular position, and the dotted line shows a threshold LOD for statistical significance.

Variation in plant height among MAGIC lines was largely related to the presence of the *erecta* dwarfing allele (figure 1). We identified 33 (out of 484) MAGIC lines as carriers of the *erecta* allele, and these lines were on average 17.3 cm shorter than other lines in isolation (95% CI: 15.5, 19.0) and 15.2 cm shorter than other lines in groups (95% CI: 13.7, 16.7) (figure 1A). In both the isolation and group treatments, variation in height mapped to a locus on Chromosome 2, near to the ERECTA gene at 11.21 Mb (figure 1B; table 1).

**Table 1.** Statistically significant QTLs in this study.

trait (dataset)	peak position (chromosome, Mb)	SNP name	LOD	95% **CI** (lower, upper Mb)		*R* ^2^
height (full)	2, 11.324	MASC06116	59.38	11.209	11.324	0.43
height (reduced)	2, 11.324	MASC06116	59.36	11.200	11.480	0.42
RII_silique no._ (reduced)	4, 1.932	MASC02074	10.63	1.554	2.700	0.13
	4, 0.701	MASC02680	10.62	0.429	1.132	0.13
RC_silique no._ (reduced)	4, 0.701 4, 1.932	MASC02680 MASC02074	10.74 10.19	0.428 1.553	2.803 2.803	0.13 0.12

QTL peaks detected in the MAGIC experiment, with the nearest single nucleotide polymorphism (SNP) and proportion of explained variance (*R*^2^) in plant height, the relative interaction intensity (RII_silique no._) and response to competition (RC_silique no._). QTLs for plant height are from the group treatment only, but we note that QTLs for plant height in isolation were nearly identical.

#### (ii) The *erecta* allele promotes group productivity

We found some evidence that the *erecta* allele promoted group productivity in the MAGIC lines. Lines with *erecta* produced on average 42.4 (95% CI: 32.5, 52.3) more total siliques in the group treatment than they did in isolation. In contrast, this average difference in all other MAGIC lines was only 31.3 siliques (95% CI: 28.2, 34.3).

**Figure 2.**
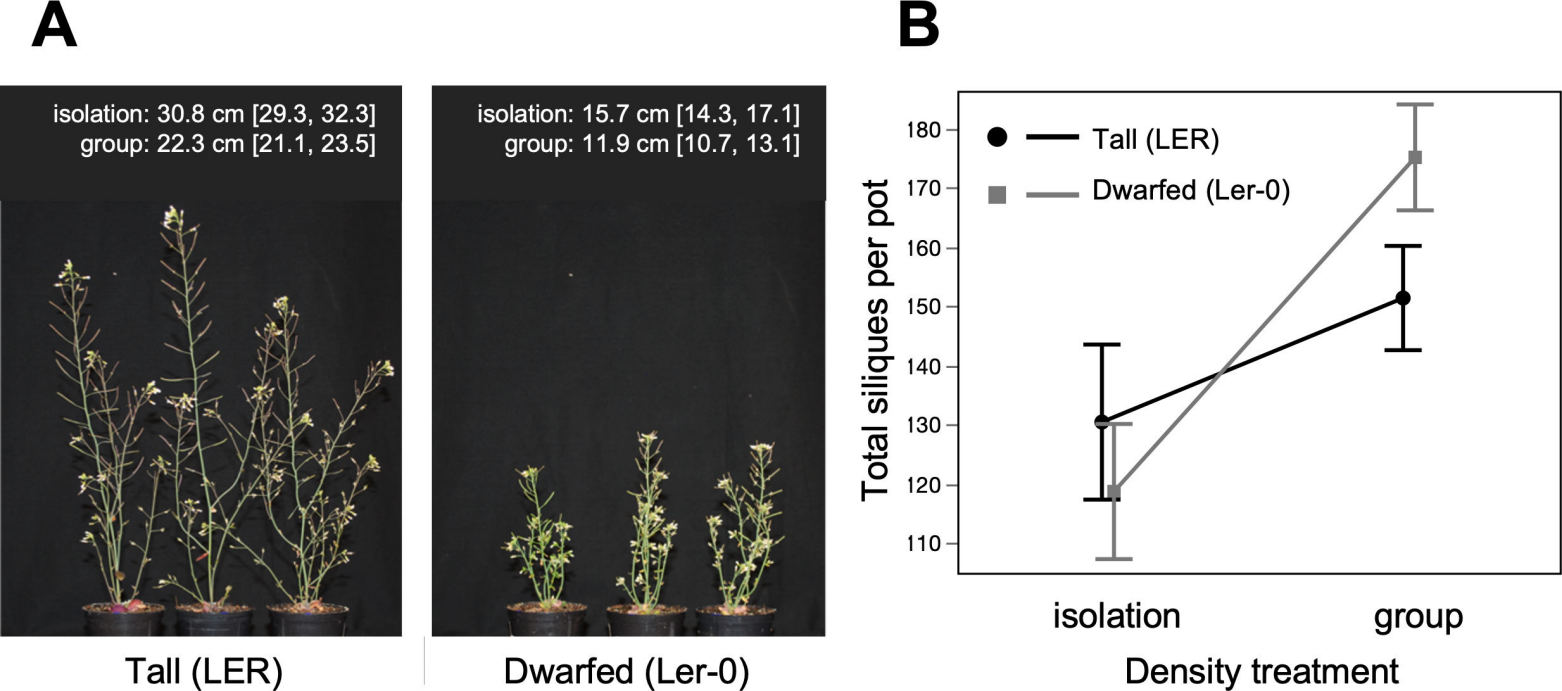
The *erecta* dwarfing allele promotes group productivity. (A) Least-squares mean plant heights for tall (LER) and dwarfed (Ler-0) isogenic lines, with representative examples pictured in the isolation treatment. (B) Total silique yields of tall and dwarfed lines were similar in isolation, but the dwarfed line was more productive, on average, in groups (error bars are 95% confidence intervals).

In the separate ERECTA experiment (using isogenic tall and dwarfed lines), we confirmed that the *erecta* allele had differential effects on individual and group productivity (figure 2). In the isolation treatment, there was no significant difference in silique production between tall (LER) and dwarfed (Ler-0) genotypes, although dwarfed genotypes produced fewer siliques, on average (mean difference of 12.9 siliques [95% CI: −9.9, 35.8]). In contrast, groups of dwarfed plants produced significantly more total siliques than groups of tall plants (mean difference of 23.5 siliques [95% CI: 7.0, 39.9]). The two genotypes had similar numbers of seeds/silique in groups, suggesting that dwarfed groups also produced higher total seed yields than tall groups (Ler-0: 45.4 seeds/silique [95% CI: 42.3, 48.6]; LER: 46.5 seeds/silique [95% CI: 43.3, 49.6]). Overall, these results imply that the dwarfed (Ler-0) genotype was less competitive than the tall (LER) genotype.

### (b) Generic measures of individual competitiveness

#### (i) Variation in competitiveness beyond the effects of dwarfing

**Figure 3.**
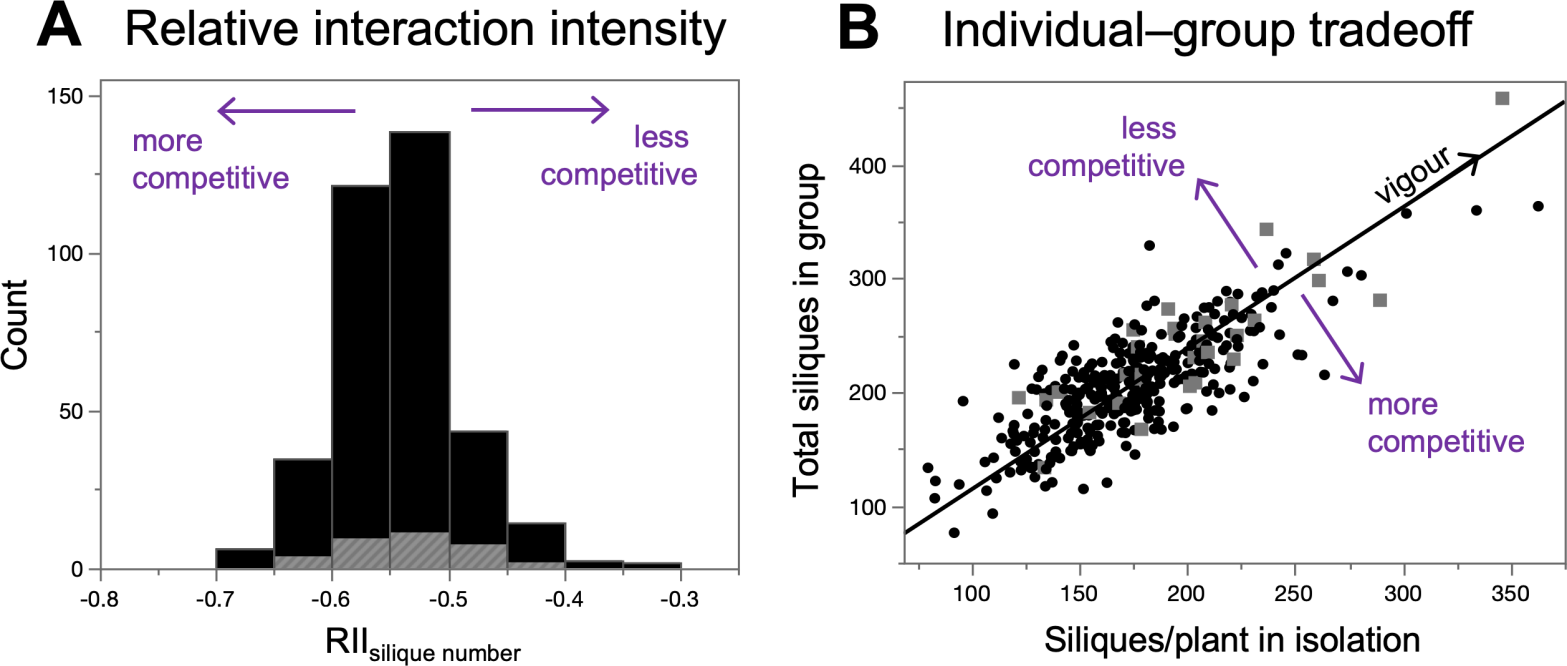
Variation among MAGIC lines in generic measures of individual competitiveness. In both panels, grey data points indicate MAGIC lines with the *erecta* mutation (*n* = 31 out of 361 lines). (A) More negative values of the relative interaction intensity (RII_silique no._) indicate more competitiveness. (B) The individual-group tradeoff measures orthogonal deviations from the regression line, where deviations below the line (negative tradeoff scores) indicate more competitiveness.

Using generic measures of individual competitiveness, we found wide variation among MAGIC lines that extended beyond the effects of the *erecta* allele (figure 3).

The relative interaction intensity (RII_silique no._) was negative for all MAGIC lines, implying that interactions in the group treatment were always competitive (figure 3A). Excluding lines carrying *erecta*, the RII_silique no._ varied widely with a mean of −0.54 (95% CI: −0.52, −0.55) and a range from −0.69 to −0.33 (most competitive to least competitive). In comparison, the mean RII_silique no._ for MAGIC lines with the *erecta* allele was −0.53 (95% CI: −0.55, −0.51), and these lines ranged from −0.62 to −0.43.

In the individual-group tradeoff analysis, we found the expected positive regression between the number of siliques/plant in isolation and total silique production in groups (figure 3B). MAGIC lines without *erecta* varied widely in their orthogonal distance from the line (tradeoff score), with a mean of −0.13 (95% CI: −1.1, 0.19) and a range from −31.3 to 34.4 (most competitive to least competitive). In comparison, the mean tradeoff score for MAGIC lines with the *erecta* allele was 1.37 (95% CI: −2.17, 4.92), and these lines ranged from −21.0 to 18.1.

Generic measures of competitiveness were correlated with plant height, even after removing the effects of the *erecta* allele. After excluding MAGIC lines carrying *erecta*, the LS mean plant height of a line in isolation was negatively correlated with the RII (*r* = −0.25; *p* < 0.0001) and individual-group tradeoff scores (*r* = −0.26; *p* < 0.0001). This means that the tallest MAGIC lines in isolation tended to be more competitive than shorter lines in isolation.

#### (ii) Genetic basis of competitiveness in general

**Figure 4.**
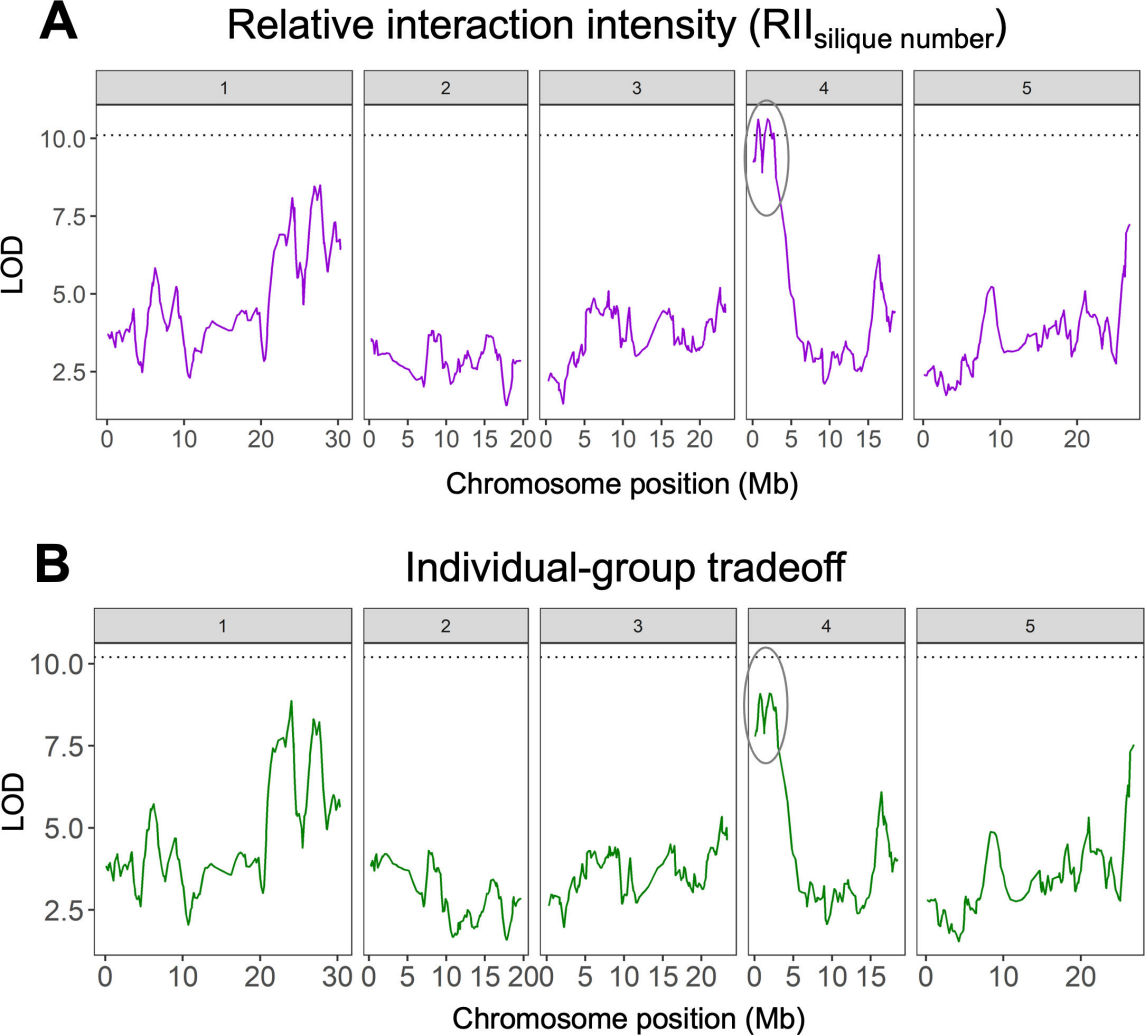
Genetic basis of individual competitiveness in the MAGIC population. (A) QTL map for the relative interaction intensity (RII_silique no._), showing significant peaks at Chromosome 4 (circled). (B) QTL map for individual-group tradeoff scores, showing notable but statistically non-significant peaks in the same region. The LOD score measures the strength of evidence for a QTL at a particular position, and the dotted line shows a threshold LOD for statistical significance.

We found novel QTLs underlying variation among MAGIC lines in generic measures of competitiveness. On Chromosome 4, we found significant QTLs underlying variation in RII_silique no._ and RC_silique no._ (figure 4A; table 1). In the same region, we also found notable but statistically non-significant QTLs underlying variation in individual-group tradeoff scores (figure 4B). The QTLs on Chromosome 4 were generally weaker and less precise than the QTL for plant height. This was not simply due to the greater number of lines used to map plant height, as a relatively precise QTL for height was detected with the reduced dataset as well (table 1).

#### (iii) Comparing different measures of competitiveness

**Figure 5.**
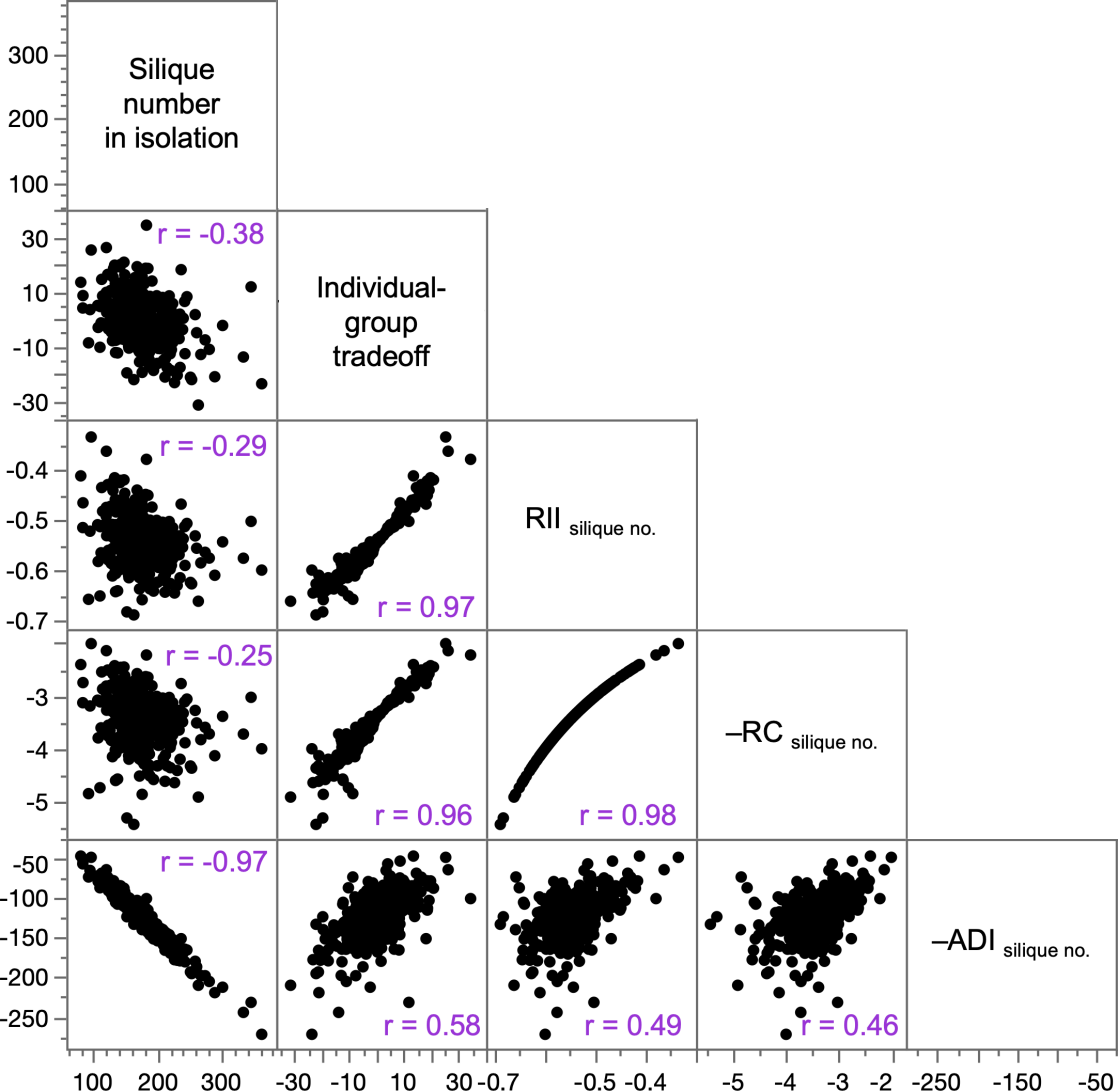
Correlations among generic measures of competitiveness and silique production in isolation. Each data point is a MAGIC line (*n* = 361 lines). Formulas for competition indices, in terms of silique number/plant in the isolation treatment and group treatment (*S*_I_ and *S*_G_, respectively): RII _silique no._ = (*S*_G_ – *S*_I_)/(*S*_G_+*S*_I_); RC_silique no._ = *S*_I_ / *S*_G_; ADI_silique no._ = *S*_I_ – *S*_G_.

Not surprisingly, we found that different generic measures of competitiveness were positively correlated, so MAGIC lines that were relatively competitive by one measure tended to be relatively competitive by other measures (figure 5). However, the weakest correlations involved the adaptation to density index (ADI_silique no._). Moreover, whereas most measures of competitiveness were only moderately correlated with silique number in isolation (−0.25 > *r* > −0.38)—showing that highly productive lines in isolation tended to have higher measures of competitiveness—ADI_silique no_ was almost perfectly correlated with silique production in isolation (*r* = −0.97). This means that the ADI provided little information about competitiveness beyond reflecting variation in silique production in isolation (electronic supplementary material, figure S5).

## 4. Discussion

Using the *A. thaliana* MAGIC population, we compared different methods for uncovering the genetic basis of plant competitiveness. Starting with an obvious social trait, we found that a major dwarfing gene, the *erecta* allele on Chromosome 2, caused reduced competitiveness and higher group productivity (figure 2). We then used the MAGIC lines to measure competitiveness more generally, based only on their productivity in isolation and in groups. We found (i) extensive variation in generic measures of competitiveness that extended beyond the effects of the *erecta* allele (figure 3); (ii) a novel genomic region, on Chromosome 4, underlying variation in competitiveness (figure 4); and (iii) that some measures of competitiveness were more useful than others (figure 5).

### (a) Comparing a major dwarfing gene to competitiveness in general

We found that it was relatively easy to map the genetic basis of an obvious social trait. We mapped plant height to a small genomic region near the ERECTA gene, consistent with previous studies of the MAGIC population [18]. This allowed us to proceed directly to isolating the *erecta* mutation as a major dwarfing gene that can promote group productivity. The *erecta* allele is known for diverse consequences on plant development that are mediated by effects on auxin hormones [23–25]. In this way, *erecta* is similar to dwarfing genes of the Green Revolution, which act through effects on gibberellin hormones [26].

The mechanism by which the *erecta* allele benefits *Arabidopsis* groups is still unclear. The group benefit of Green Revolution genes in wheat and rice crops may be partly explained by reduced shading among dwarfed (shortened) plants. In *Arabidopsis*, however, shading is more likely when plants grow as leafy rosettes near to the ground [27]. The group benefit of *erecta* may therefore be unrelated to plant height *per se*. Moreover, it is notable that dwarfed *erecta* plants in isolation did not have higher silique production than isogenic tall plants in isolation (figure 2B), implying that the benefit of *erecta* is not owing to a reallocation of resources from stem production to silique production. Instead, the group benefit of *erecta* may involve reduced shading at the rosette stage, where the *erecta* mutation is associated with more compact rosettes [28], or reduced competition over soil resources. Whatever the mechanism, our results suggest that *erecta* mutants could be tested more widely as candidate genes for reduced competitiveness in high-density crops.

Beyond the effects of the *erecta* allele, the *Arabidopsis* MAGIC population contained extensive variation in competitiveness more generally. Several other studies using genetically diverse plant populations have also found wide variation in generic measures of competitiveness [15–17,20]. Our results, however, show that genetic variation in competitiveness extended beyond the effects of a major dwarfing mutation. In fact, MAGIC lines with the *erecta* allele had relatively moderate competitiveness compared to other MAGIC lines (which helps to explain why we did not detect a competitiveness QTL at the ERECTA locus). This suggests that the MAGIC population has great potential for discovering novel genes with relatively large effects on plant competitiveness.

We mapped generic measures of competitiveness to a single genomic region on Chromosome 4 of *A. thaliana*, suggesting the possibility of a novel gene (or genes) for reduced competitiveness. Our QTL analyses indicated two peaks lying within candidate genes of interest: AT4G04020.1, a fibrillin precursor protein; and AT4G01630, a member of the Alpha-Expansin gene family with known effects on lateral root formation [29]. This potential link between competitiveness and root phenotypes is consistent with a previous study of *A. thaliana*, which found a gene on Chromosome 3 for reduced root allocation and lower competitiveness [17]. Smaller roots may cause reduced competitiveness and smaller plant size in general, which could also explain why the least competitive MAGIC lines in our experiment tended to be relatively short.

It was surprising to detect only a single genomic region underlying generic measures of competitiveness, though this is consistent with previous genetic studies of plant–plant interactions [15,17,30]. One explanation is that—contrary to expectations of a complex quantitative trait—plant–plant interactions are often influenced by major genes with large effects, rather than many genes with small effects. On the other hand, the QTLs that we detected for generic measures of competitiveness were much less precise than the major-effect QTL for plant height. It may be that competitiveness is influenced by many loci with small effects, and we could detect only the largest of these. Experiments with greater power (e.g. more genotypes and more genetic markers) may be necessary to detect additional loci underlying variation in competitiveness (and plant–plant interactions, more generally).

### (b) Comparing different measures of competitiveness

We found differences among generic measures of competitiveness that made some more informative than others. A useful measure of competitiveness should account for the fact that some genotypes will grow better in the experimental environment than others (genotypes will vary in their overall vigour in the environment). Competition indices do this by calculating a relative measure of competitiveness—in our experiment, based on a genotype’s productivity in monoculture groups and in isolation. However, it is important to check that competition indices combine information about individual and group productivity as expected. We found that the ADI (from [15]) mostly reflected productivity in isolation (not competitiveness *per se*), making it less useful than other indices (RII or RC).

The individual-group tradeoff approach is perhaps the most natural method for measuring competitiveness in a way that is independent of vigour (figure 3B). We assessed this tradeoff by measuring individual performance in isolation (no competition), whereas others have measured individual performance in mixed-genotype groups (inter-genotypic competition) [17,31]. It is possible that these two alternative methods measure something fundamentally different. However, we propose that a genotype’s productivity in isolation (i.e. a plant’s ability to gather resources for itself) will usually predict its productivity in mixed-genotype groups, making the two methods largely similar. Moreover, a key advantage of our method is that plant breeders will probably find it easier to replicate genotypes in isolation rather than in mixed-genotype groups.

## 5. Conclusions

In many ways, our results reflect the history and future challenges of plant breeding and agriculture. Similar to advances during the Green Revolution, we found that it was relatively easy to take advantage of a major-effect dwarfing gene to boost group productivity in *Arabidopsis.* It remains a greater challenge to precisely identify the remaining genes underlying reduced competitiveness more generally. Yet, our results suggest that modern genomic resources, and especially the growing number of multi-parent populations in crops [32], should be excellent tools for addressing this challenge. Plant breeders could use these populations and adopt the methods of our approach to find novel genes for more cooperative crops.

## Data Availability

Our data can be accessed via the Dryad data repository [33]. Supplementary material is available online [34].
